# Foraging Responses of Black-Legged Kittiwakes to Prolonged Food-Shortages around Colonies on the Bering Sea Shelf

**DOI:** 10.1371/journal.pone.0092520

**Published:** 2014-03-26

**Authors:** Rosana Paredes, Rachael A. Orben, Robert M. Suryan, David B. Irons, Daniel D. Roby, Ann M. A. Harding, Rebecca C. Young, Kelly Benoit-Bird, Carol Ladd, Heather Renner, Scott Heppell, Richard A. Phillips, Alexander Kitaysky

**Affiliations:** 1 Department of Fisheries and Wildlife, Oregon State University, Corvallis, Oregon, United States of America; 2 Ocean Sciences Department, University of California Santa Cruz, Santa Cruz, California, United States of America; 3 Department of Fisheries and Wildlife, Oregon State University, Hatfield Marine Science Center, Newport, Oregon, United States of America; 4 U.S. Fish and Wildlife Service, Anchorage, Alaska, United States of America; 5 U.S. Geological Survey-Oregon Cooperative Fish and Wildlife Research Unit, Oregon State University, Corvallis, Oregon, United States of America; 6 Environmental Science Department, Alaska Pacific University, Anchorage, Alaska, United States of America; 7 Institute of Arctic Biology, University of Alaska Fairbanks, Fairbanks, Alaska, United States of America; 8 College of Earth, Ocean, and Atmospheric Sciences, Oregon State University, Corvallis, Oregon, United States of America; 9 Pacific Marine Environmental Laboratory, National Oceanic and Atmospheric Administration, Seattle, Washington, United States of America; 10 Alaska Maritime National Wildlife Refuge, U.S. Fish and Wildlife Service, Homer, Alaska, United States of America; 11 British Antarctic Survey, Natural Environment Research Council, Cambridge, United Kingdom; Norwegian Polar Institute, Norway

## Abstract

We hypothesized that changes in southeastern Bering Sea foraging conditions for black-legged kittiwakes (*Rissa tridactyla*) have caused shifts in habitat use with direct implications for population trends. To test this, we compared at-sea distribution, breeding performance, and nutritional stress of kittiwakes in three years (2008–2010) at two sites in the Pribilof Islands, where the population has either declined (St. Paul) or remained stable (St. George). Foraging conditions were assessed from changes in (1) bird diets, (2) the biomass and distribution of juvenile pollock (*Theragra chalcogramma*) in 2008 and 2009, and (3) eddy kinetic energy (EKE; considered to be a proxy for oceanic prey availability). In years when biomass of juvenile pollock was low and patchily distributed in shelf regions, kittiwake diets included little or no neritic prey and a much higher occurrence of oceanic prey (e.g. myctophids). Birds from both islands foraged on the nearby shelves, or made substantially longer-distance trips overnight to the basin. Here, feeding was more nocturnal and crepuscular than on the shelf, and often occurred near anticyclonic, or inside cyclonic eddies. As expected from colony location, birds from St. Paul used neritic waters more frequently, whereas birds from St. George typically foraged in oceanic waters. Despite these distinctive foraging patterns, there were no significant differences between colonies in chick feeding rates or fledging success. High EKE in 2010 coincided with a 63% increase in use of the basin by birds from St. Paul compared with 2008 when EKE was low. Nonetheless, adult nutritional stress, which was relatively high across years at both colonies, peaked in birds from St. Paul in 2010. Diminishing food resources in nearby shelf habitats may have contributed to kittiwake population declines at St Paul, possibly driven by increased adult mortality or breeding desertion due to high foraging effort and nutritional stress.

## Introduction

Dynamics of seabird populations reflect changes in adult survival, offspring production and recruitment, and are strongly affected by disruptions in their food supplies due to changes in climate and oceanographic conditions [40], [44], and human exploitation [27], [18]. Continental shelves are hot spots of global importance for both seabirds and fisheries targeting forage and other fish [47]. Ocean basins are often characterized by ephemeral areas of enhanced productivity, such as mesoscale eddies, that concentrate micronekton (crustaceans, cephalopods, fish) [78], [55], [3], [31], and support large communities of top predators [62], [16], [82], [63]. Because foraging seabirds can travel across different oceanographic domains [91], [30], to understand the causes of population changes and to identify regions of conservation concern, it is critical to identify and characterize the use of different habitats. This is now possible with the combined use of miniaturized electronic tags [11], [34], remote-sensed oceanographic data [9], [85], concurrent measures of seabird diets [62], [66], and measures of at-sea prey biomass and distribution [6].

The spatial distribution of seabirds is affected by changes in abundance [39], patchiness [6], and persistency of prey in resource hot spots [79]. Seabirds are known to increase foraging ranges in response to reductions in prey availability [83], [8], and to adopt behavioral strategies (e.g. relative reliance on day or night, or short or long trips; often reflected in a dietary shift) to balance adult and chick needs [14], [66]. Seabird distribution is also influenced by the diurnal or nocturnal availability of their prey; Cory ‘s shearwaters (*Calonectris diomedea*) forage more often at night in basin than shelf regions apparently due to differences in abundance of vertically migrating prey [20]. In addition to bottom-up processes, intra- and inter-specific competition for food could also affect seabird distribution through a variety of density-dependent mechanisms [2], [77]. These predictions are supported by evidence of segregation of foraging areas between conspecifics from neighboring colonies [36], [97], [89], and of greater foraging ranges of birds from larger colonies [54], [1]. Sympatric seabird species feeding on the same prey have been shown to overlap in feeding areas in productive systems [92], but to show spatial and temporal segregation elsewhere [33], [61].

Long- lived animals such as seabirds are predicted to limit current investment in reproduction if it is likely to reduce survival and have a negative impact on lifetime reproductive output [22], [15]. Support for this prediction is, however, equivocal, with some studies showing that parents prioritize their own survival over success in the current reproductive attempt [69], [60], and others showing that they will accept some long-term costs [67], [65], [74]. Experimental studies have demonstrated flexibility in reproductive investment in some seabirds [70]; and that in stochastic environments they adjust their breeding effort according to both their own body condition and the needs of the offspring [25]. Costs associated with raising chicks during food shortages in black-legged kittiwakes (*Rissa tridactyla*) include reduced fledging success [65], [84], [66], lower adult body condition [32], [50], elevated levels of physiological stress (measured by baseline corticosterone [CORT]) [49], [50], and reduced survival [32], [65]. CORT plays an important role in an individual’s adaptive response to environmental stress [94], [72]; levels have been shown to be elevated during food shortages [48], [49], [50], under conditions of experimentally increased foraging effort [37], and in individuals in poor body condition [48], [71]. Given the relationship between high stress levels and low return rates of breeding kittiwakes [50], [35], CORT levels have been used as a proxy of adult survival in population modeling [75].

In recent decades, the eastern Bering Sea Shelf has shown considerable variability in climatic conditions [43], [81]. Populations of black-legged kittiwakes breeding at the Pribilof Islands have shown divergent trajectories; a decline at St. Paul since 1975, but no long-term trend at St. George [12]. Concurrent with the population decline at St. Paul, there has been a decrease in juvenile pollock (*Theragra chalcogramma*) and increase in mesopelagic myctophids in kittiwake diets [80], [68]. In 2009, tracked kittiwakes obtained myctophids solely in the basin [66]. Thus, changes in conditions in continental shelf waters might have required increased foraging effort by kittiwakes forced to switch to oceanic habitats, affecting breeding success or adult survival. Given that St. Paul is 3-times farther from the shelf break than St. George, we expect a stronger effect of location on kittiwakes at this colony [12], [66]. On the other hand, intra- and inter-specific competition for food is likely to be higher at St. George given the larger population [42], and substantially higher number of red-legged kittiwakes (*Rissa brevirostris*), which feed mainly on myctophids [80]. To test these predictions, we analyzed tracking, and diet data collected from kittiwakes at both colonies during three cold years, when abundance of juvenile pollock was low on the shelf [41]. We also examined association of foraging kittiwakes with basin eddies, which influence the distribution of northern fur seal (*Callorhinus ursinus*) in the southeastern Bering Sea [82], [63], and used eddy kinetic energy as a proxy for oceanic prey availability [96], [21], [19].

Relative fitness consequences of different foraging strategies were assessed on the basis of chick feeding rates and fledging success (nests with fledglings/nests with chicks) [12]. Baseline CORT levels were used to measure nutritional status of adults, which is linked to subsequent survival probability [50], [35]. The influence of the study factors on prey availability in shelf and basin habitats, the observed effects on predators and prey, and expected responses of black-legged kittiwakes nesting at the Pribilof islands is summarized in Table 1. We use these outcomes to propose a mechanism underlying the contrasting population trajectories of the study colonies, and make predictions in the light of forecasted changes in abundance of juvenile pollock in the southeastern Bering Sea shelf [58].

**Table 1:** Summary of factors affecting prey availability in the southeastern Bering Sea shelf and basin, observed effects on predators and prey, and expected responses of black-legged kittiwakes breeding at the Pribilof islands (St. Paul & St. George).

Factors	Effects	Expectations
**1. Habitat**		
1.1 Shelf: Food webs affected by cold and warm regimes (1)	In cold years: -Lower abundance and dispersed distribution of juvenile pollock and capelin compared to warm years (2) -Low food availability for piscivorous seabirds (3)	During three cold years (2008-10): - Low biomass of juvenile pollock on shelf (Y) & occurrence of shelf-based prey in bird diets (Y) - High foraging effort and nutritional stress incurred by kittiwakes (Y)
1.2 Basin: Food webs influenced by eddy currents near the Pribilof Islands	- Concentrations of mesopelagic prey increase in areas of high eddy kinetic energy (EKE; 4, 5, 6)	During high-EKE years: - Kittiwakes forage near eddies (Y) - Higher prevalence of oceanic prey (e.g., myctophids) in diets (Y) - Higher incidence of kittiwake foraging trips to the basin (Y) - Higher kittiwake fledging success (Y)
**2. Colony**		
2.1 Proximity of seabird colony from alternative foraging habitats in Basin (St. Paul > St. George)	- Lower ability to cope with food shortages in vicinity of St. Paul (7, 8)	St. Paul compared to St. George: - Higher kittiwake usage of shelf habitats (Y) - Longer foraging trips to basin (Y) - Higher nutritional stress (P) - Lower fledging success (N)
2.2 Colony size and population size of red-legged kittiwakes (main prey is myctophids) (St. George > St. Paul)	- Higher density-dependent feedback on seabird populations on St. George (9)	St. George compared to St. Paul: - Longer trips to shelf & basin (N) - Lower myctophid occurrences in diets (N) - Higher nutritional stress (N)

Support for expectations shown in parentheses (Y =  yes, N = no, P = partial).

Stabeno et al. 2012 (1); Hollowed et al. 2012 (2); Satterthwaite et al. 2012 (3); Zainuddin et al. 2006 (4), Drazen et al. 2011 (5); Dell et al. 2011(6); Byrd et al. 2008 (7); Paredes et al. 2012 (8), Hunt 1986 (9).

## Methods

### Ethics Statement

All research was conducted in accordance with the Animal Care and Use Committees of the respective institutions of the author responsible for those data, and complied with all applicable laws. Stomach contents were collected in accordance with the American Fisheries Society’s Guidelines for the Care and Use of Fish in Research and the Institutional Animal Care and Use Committee of Oregon State University (permit 3659). Seabird cliffs on the Pribilof Islands are part of the Alaska Maritime National Wildlife Refuge. Seabirds were studied in a collaborative effort with Refuge staff (permit 20570), following the United States Government Principles for the Utilization and Care of Vertebrate Animals and the Animal Care Committee of the United States Fish and Wildlife Service (permit 200908).

### Study System

We studied black-legged kittiwakes (hereafter kittiwakes) at two Pribilof Islands, St. Paul (104 km^2^, 57°7’N 170°17’W) and St. George (90 km^2^, 56°36’N 169°33’W), located on the continental shelf of the southeastern Bering Sea, Alaska. Compared to St. George, St. Paul is three times farther (∼ 90 km) from the shelf break and oceanic habitats where basin eddies contribute to the high primary productivity [78], [57]. Our study focused on two distinct marine habitats with different bathymetric regimes, the shelf (100–200 m depth) and the basin (> 200 m depth), and the associated food webs. Some pelagic prey, such as the juvenile pollock, undertakes diel vertical migration on the continental shelf and can be found at <10 m depth [76], [6]. In the Bering Sea basin, the most abundant myctophid species *Stenobrachius leucopsarus* is mostly found in deep waters (500–700 m depth) during the day, and makes a vertical migration to the surface at night [4]. Other myctophids such as *S. nannochir* and *Nannobrachium regale* are non-migrants; they remain at 400–700 m depth during day and night [29], [90]. The bioluminescent organs of myctophids (in the body and head region) increase their susceptibility to capture by visual predators during darkness [88].

Kittiwakes are monogamous, with both parents incubating the eggs and feeding the chicks, and have slight sexual size dimorphism with males larger and heavier than females [46]. During the chick-rearing period, kittiwakes feed mainly on fish captured by plunge diving (< 1 m). Their main forage fish species at the Pribilof Islands, in increasing order of energy content [93], are juvenile pollock, Pacific sandlance (*Ammodytes hexapterus*) and myctophids [80], [68]. Byrd et al. (2008) estimated a total of 15,000 and 1,600 individual black- and red-legged kittiwakes respectively at St. Paul; and 72,000 and 220,000 respectively at St. George in 2005. Nest predation for birds on ledges at both islands is minimal [12].

### Field-data Collection

Data on kittiwake foraging trips, diets, chick-feeding frequency, fledging success and nutritional stress were collected simultaneously at both Pribilof Islands in 2008, 2009 and 2010. Most field activities were undertaken during the chick-rearing period, from the last week of June to the first week of August. In total, 155 adult kittiwakes with chicks aged 5–25 days were captured at their nests using 8-m noose poles and snare traps. GPS data loggers (GiPSy 2 and 3 - Technosmart; flat antenna with 250 mA battery; size  =  41×14×7 mm; weight  =  10 – 12 g) were attached to the dorsal surface of four central retrices using white Tesa tape #4651. The GPS loggers were encapsulated in a shrink tubing (2 g; 4FT IC8725 ¾ inches clear; Frigid North, AK, USA) prior to deployment to ensure waterproofing. We initially set a GPS recording interval of 1 s to provide higher-resolution tracking data, but that meant that coverage of some trips was incomplete, especially those to the basin. Latterly, most devices were set to record at 180 s intervals to try and ensure the battery would last for the longer trips. The tracking data have been deposited in the Bering Sea Data Archive: http://beringsea.eol.ucar.edu/. Activity loggers, carrying saltwater immersion sensors (British Antarctic Survey; Mk13  =  1.8 g; MK9  =  2.5 g), attached to plastic color bands were deployed simultaneously on all birds with GPS loggers for determination of feeding behavior. Activity loggers were set to record wet and dry stages at a 1 or 3 s sampling interval. Total instrument weight was 3.4% of average kittiwake body mass at the two colonies (416**±**2.0 g, *n* = 292). No effect of instrumentation was found on the trip duration and stress levels of kittiwakes from the two colonies in 2009 [66].

Recapture effort started 2 days after deployment; the majority of birds (90%, *n* = 161) were recaptured within 2–4 days, and some after 5–17 days (10%). At first capture, blood was collected for CORT analysis (see below), birds were weighed using a 500 g Pesola balance (±5 g), banded with a USFWS metal band, fitted with loggers, and temporarily marked with livestock paint to facilitate individual identification. Upon recapture, devices were retrieved, birds were weighed, and diet and blood samples collected. Blood samples (< 100 μl) were collected from the brachial vein within three minutes of capture according to a standardized technique for CORT analysis [7], and for sex determination [28]. Samples were centrifuged, and plasma and red cells were preserved frozen for later laboratory analysis at the University of Alaska Fairbanks.

Diet samples were obtained from opportunistic regurgitations of captured adults or chicks, and in 2008 the stomach contents of adults were obtained using water offloading [66]. All sample types were pooled for analysis. Additional samples were obtained from untagged birds to increase sample sizes (St. Paul *n* = 58, St. George *n* = 51). Diet samples were weighed using a 100 g balance (±1 g) immediately after collection and preserved with 70% ethanol for subsequent laboratory analysis. For some analyses, diet samples obtained during this study were incorporated into the longer-term time series of diet studies at each colony (1993–2010) [68].

Monitoring of kittiwake fledging success was initiated prior to chick hatching in late June and concluded at fledging in late August. Yearly, on each island, 6–17 plots of kittiwakes containing 7–45 nests, were monitored every 3–5 days to determine fledging success (51, 86]. A subset of nests (*n* = 10–12 per island) with un-instrumented birds was observed from dawn to dusk (0600–2330h) to determine chick-feeding frequencies during 3–7 days at both colonies in 2009 and 2010. Observations were conducted during early, mid, and late chick rearing to account for any difference in provisioning behavior associated with chick age. Historical data on kittiwake diet and productivity used in this study are available from the Alaska Maritime National Wildlife Refuge website: http://www.fws.gov/alaska/nwr/akmar/whatwedo/bioprojects/publications.htm.

Details of the collection and analysis of data on at-sea abundance of prey can be found in Benoit-Bird et al. [5]. Briefly, ship-based sampling of the environment and potential prey took place in a 200 km radius of St. Paul and St. George Island from mid July to mid August in 2008 and 2009, along 10-km long transects according to a stratified random design. Multi-frequency acoustic data were combined with targeted trawls to estimate the distribution of potential prey around the colonies. 38 kHz integrated acoustic scattering identified juvenile pollock, and was combined with the target strength-weight relationship for pollock in the Bering Sea [87] to estimate the average biomass density (g/m^2^) of pollock for each transect, apportioned to either age-0 or age-1+ pollock using trawl data.

### Eddy Kinetic Energy as a Proxy for Oceanic Prey Abundance

Myctophid fishes, the main basin-associated forage fish of kittiwakes at the Pribilof Islands in recent years, have been found in elevated concentrations near fronts and mesoscale eddies [55], [73], [59]. Other oceanic prey, such as large zooplankton, has also been found in higher biomass on edges of anticyclonic eddies than in open water [31]. We used eddy kinetic energy (EKE) [96], [21], [19] as a proxy of the availability of oceanic prey (particularly myctophids). Based on published studies, we assumed that the absence of a positive relationship between EKE and the occurrence of pelagic prey in kittiwake diets indicates no influence of eddy activity on prey acquisition. Thus, we used a one-tailed test; our null hypothesis was that there is no positive relationship between EKE and oceanic prey (myctophid, squid, euphausiid or amphipod) occurrence in kittiwake diets; the alternative hypothesis is that there is a positive relationship between these variables. Gridded sea-surface height anomalies (SSHA) from AVISO (Archiving, Validation, and Interpretation of Satellite Oceanographic Data) were used to calculate EKE, as a measure of annual mesoscale variability during the summer [53]. The AVISO “ref merged” dataset (from http://www.jason.oceanobs.com) consists of delayed-mode, merged data from two satellite missions, Topex/Poseidon and ERS followed by Jason-1 and Envisat. The mapped altimetry data set includes one map every 7 days with a 1/3° spatial resolution on a Mercator grid [23].

Assuming geostrophy, EKE (per unit mass) is estimated from SSHA (η*'*) as:
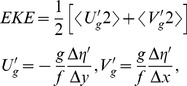



where *U'_g_* and *V'_g_* are the geostrophic velocity anomalies, *f* is the Coriolis parameter, g is gravity and *< >* denotes the time average. Δ*x* and Δ*y* are distances in longitudinal and latitudinal directions.

Mean EKE (cm^2^/s^2^) was calculated for July of each year from 1993 to 2010 (Appendix S1) within a minimum convex polygon encompassing all at-sea locations obtained from GPS tracking in the three years of the study. The percentage of occurrence of oceanic prey species and fledging success of kittiwakes on the two islands were compared with EKE values in each year, with sample sizes of 6–10 years in each correlation, including data from the three years in which kittiwakes were tracked. We excluded years (2001 at St. George, and 2003 and 2009 at St. Paul) in which myctophids accounted for < 4% of prey in diets (Appendix S1) because this indicated negligible use of basin habitats [66].

### Foraging Behavior and Eddy Association

In a previous study [66], we found that kittiwake visited the shelf throughout the day, whereas trips to the basin were longer and occurred primarily overnight. In this study, we were interested in investigating annual differences in the usage of different marine habitats (shelf: 100–200 m depth; basin: > 200 m depth) by birds from different colonies, and the effects on foraging effort. We obtained a total of 310 trips (*n* = 153, St. Paul; *n* = 157 St. George) from 161 birds (Table 2). We calculated the frequencies of trips to each habitat based on the furthest location of individual trips from the colony. If multiple trips were obtained for the same bird, these were scored to reflect relative preference for each type of habitat, e.g., a bird that made 1 trip to the basin and 2 trips to the shelf was given values of 0.33 (basin) and 0.67(shelf), indicating greater use of shelf. The few trips to the shelf break (*n* = 5 in both 2009 and 2010) were included in the category of basin. In addition, we used combined data from GPS and activity loggers to confirm feeding locations, including those in daylight *vs.* darkness, and association with eddy features (see below).

**Table 2:** Summary statistics of data-logger deployment and the total number of trips conducted by black-legged kittiwakes at the two Pribilof Islands.

	St. Paul			St. George		
	2008	2009	2010	2008	2009	2010
GPS/activity loggers deployments	22	19	35	23	23	39
Loggers recovered	21	17	35	21	21	39
Bird abandoned/nest failed	1	3	3	2	2	5
Loggers with usable data	15	14	35	11	19	39
Total number of trips	29	38	86	15	41	101

All tracking data (available at http://beringsea.eol.ucar.edu/) were linearly interpolated at 10 m intervals where there was a gap of < 30 min between consecutive locations. Tracks with larger gaps were not considered. Maximum trip distance (to the furthest point from the colony) and duration were calculated for each foraging trip according to criteria in Paredes et al. (2012). As mean maximum trip distance (range  =  1.3–464.7 km, *n* = 307) of individual kittiwakes was highly correlated with mean trip duration (0.62–66.7 h; Pearson's product moment correlation, r^2^ = 0.809, *P*<0.001), only the trip distance was used as a proxy of foraging effort. We included trip distance as the dependent variable in a Linear Mixed Model (LMM) with colony, sex and year as fixed factors, and individual as a random factor. Given the annual differences in the use of habitats of birds from each colony, the effect of habitat on trip distance was investigated separately to avoid an unbalanced model with empty cells. We included trip distance in 2010 (the only year in which there was enough data for both colonies) in a LMM with colony and habitat as the main factors and individual as a random factor. We used a threshold *P*-value of 0.025 to adjust for multiple comparisons of the same variable (trip distance).

For determination of foraging locations, the GPS tracking data were combined with activity logger data by subsampling the GPS records to one point every 10 minutes. A time lag of 8 minutes previous and 2 minutes following the activity record was chosen to allow the greatest number of matched points. GPS locations and wet periods were matched by time to determine potential foraging locations. Because activity loggers were attached to a leg band, data from birds resting on the sea surface for bathing or preening would indicate a continuously wet period. Therefore, we assumed that birds were not foraging when they were wet for > = 90% of the time in any ten-minute period if this period was also associated with a previous or later event of similarly high wet values (following [66], [6]). Kernel densities were generated from all foraging locations recorded on trips to the basin and the shelf by birds from each colony, by year, using a single smoothing factor (h) estimated using Least Squares Cross Validation (h = 14.05 km; HRT, ESRI ArcGIS). The distributions of foraging locations were collated by hour to examine possibly differences in the relative use of shelf and basin habitats in daylight or darkness.

Foraging locations were used to determine the use of eddy features in the basin by kittiwakes. Two different datasets were used to examine the influence of eddies. Gridded sea-surface height anomalies (SSHA) downloaded from AVISO (http://www.aviso.oceanobs.com) were used to estimate EKE (methods above). Eddy trajectory data (http://cioss.coas.oregonstate.edu/eddies/) [13], derived from the sea surface height fields in Version 3 (DT-2010) of the AVISO, were used to determine the association between kittiwake foraging locations and individual mesoscale eddies with lifetimes of > = 4 months. Eddies were continuously tracked and associated metrics provided at 7-day temporal and 0.25 degree spatial resolutions. Each kittiwake foraging location was matched temporally (± 3–7 days) to weekly eddy fields. We determined distance to the perimeter of the closest eddy, whether the foraging location was inside, near (< 20 km) or outside (20–200 km) the eddy, and whether the eddy was cyclonic or anticyclonic. To test spatial association with eddies we generated an equal number of normally distributed, random locations within the perimeter of the foraging locations in the basin for birds from the respective colony. We compared the frequency distributions of distance to nearest eddy perimeter of the random locations to those of foraging locations categorized as inside, near, and outside both cyclonic or anticyclonic eddies.

### Diet Composition

Prey items were identified to the lowest taxonomic level possible. Lengths of partially digested pollock were estimated from otolith size to determine age classes (see [68]). Total frequency of occurrence was calculated as the percentage of all samples with prey remains in which a particular prey item occurred. Adult and chick diets were combined, as in previous studies [80], [68], because these cannot be distinguished readily (adults store prey in their proventriculus for several hours before regurgitating it to the young). All analyses were limited to diet samples collected during the chick-rearing period (from hatching until chick fledging), as the diet is different in incubation [68], and to match the period when EKE was calculated. The prey of tracked birds was assumed to have been obtained in the furthest foraging habitat used prior to the day of recapture. Core-feeding areas of trips to the basin were mainly in oceanic waters, therefore preys of tracked birds were assumed to have been obtained in the basin. Percentages of prey species associated with each habitat were calculated according to the total number of samples of trips to each habitat.

### Fitness Measures

Chick feeding rates, calculated as number of feeds per hour at each nest, were compared between colonies and among years using LMMs, with nest included as a random factor. Fledging success (nests with fledglings/nests with chicks)[12] was averaged by plot to reduce the variance, and then analyzed using a General Linear Model, with colony and year as fixed factors. Log_10_ baseline CORT levels were used as a measure of nutritional stress, and as a proxy for adult survival probability, as values greater than 0.90 ng mL^–1^ predict a bird will not return subsequently to the colony in Cook Inlet, Alaska [50], [74]. Details of the laboratory analysis of plasma baseline CORT concentrations can be found in Kitaysky et al. [48]. CORT values were log-transformed before analysis using LMEs, to test maximum likelihoods with colony and year as fixed factors, and individual as a random factor.

Statistical analyses were carried out using PASW Statistics 18 and R (version 2.14.2). We used parametric tests (linear mixed models and general linear models) to compare groups if the residuals met the assumptions for the general linear model (homogeneity and normality). If not met, data (trip distance) were log-transformed before statistical analysis. Levels of significance were adjusted using Bonferroni correction when multiple tests using the same variable (i.e. distance) were performed. Pearson's product moment correlations were used to relate distance and duration of foraging trips, to EKE and oceanic prey occurrence. Multiple comparisons were undertaken using the post-hoc Tukey HSD test. We used chi-square tests with Yates correction for comparing frequencies and Kruskal-Wallis test to compare diet occurrences. Means were expressed ± SE. All comparisons were two-tailed except for the correlation between EKE and myctophid occurrence. Statistical differences were considered significant when *P* < 0.05 for all tests, and corrected to *P* < 0.025 for the analysis of trip distance (see above).

## Results

### Foraging Behavior

#### Habitat usage and trip distance

The majority of GPS and activity loggers deployed on chick-rearing birds were recovered (96%, *n* = 161), of which 75% had recorded sufficient data for analysis. About 10% of nests with chicks failed after logger deployment each year (Table 2). Nest failure rates of non-tagged birds during the chick-rearing period ranged from 46 to 75%.

**Figure 1:**
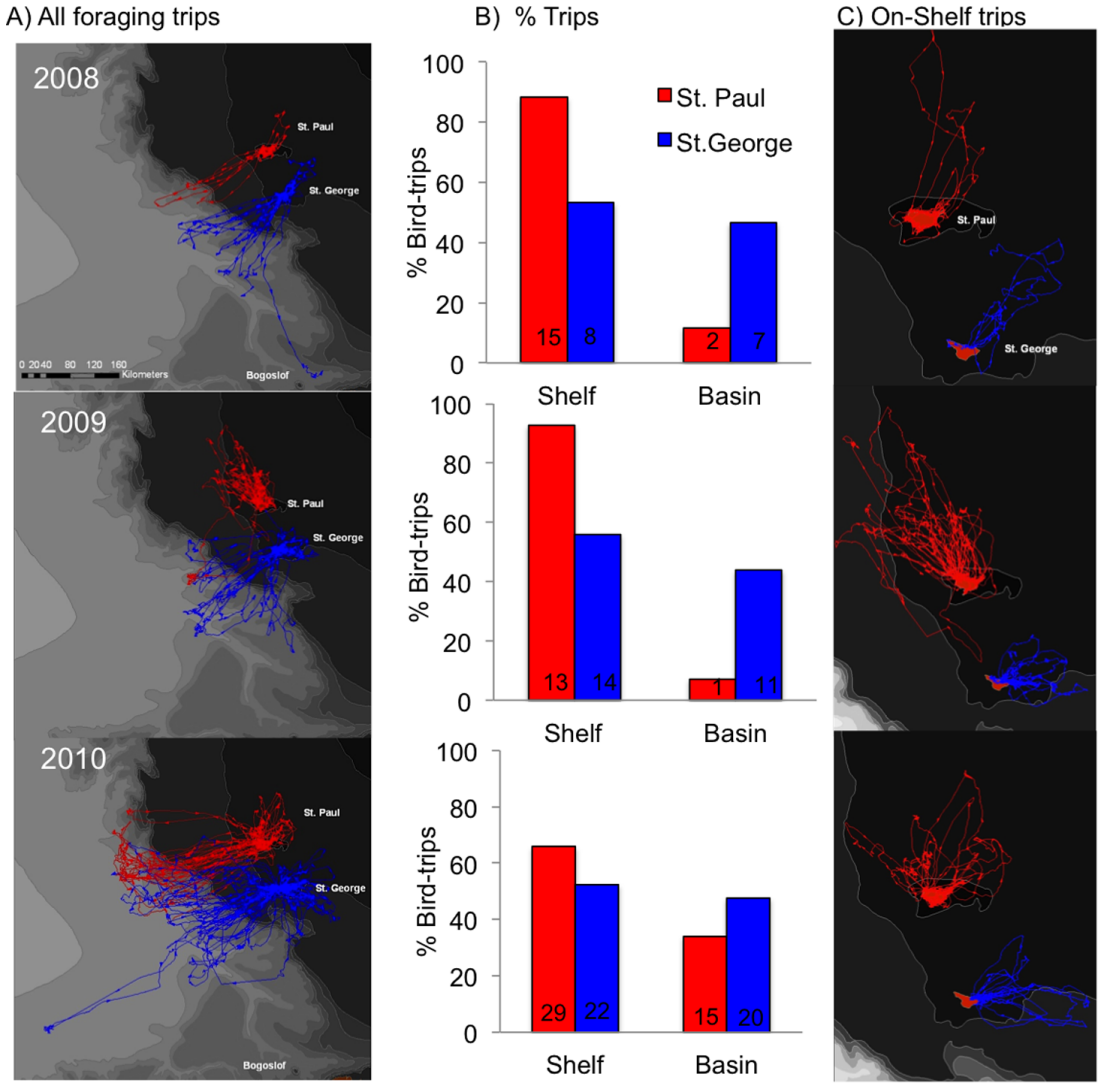
Foraging tracks of chick-rearing black-legged kittiwakes at the Pribilof Islands. A) GPS tracking (St. Paul  =  red; St. George  =  blue) during 2008 (*n* = 34 tracks), 2009 (*n* = 58) and 2010 (*n* = 70). B) Number of foraging trips to the basin or shelf habitats made by birds from St. Paul and St. George each year. C): close-ups of first maps showing nearshore (St. Paul) and shelf trips.

**Figure 2:**
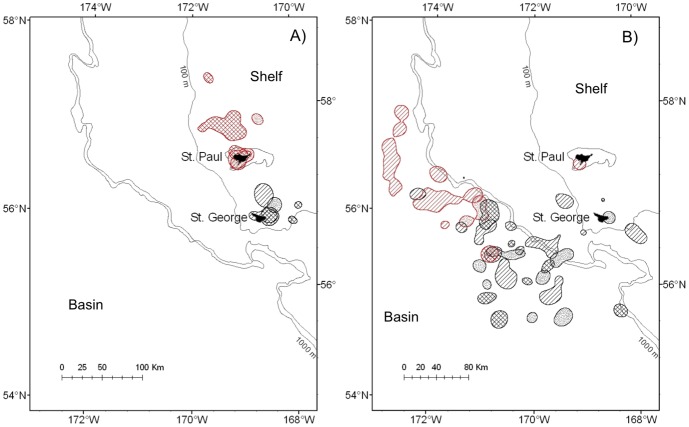
Core feeding areas (50% contours of foraging locations) of black-legged kittiwakes from St. Paul and St. George Islands during trips to marine habitats. Trips to the shelf (A), and to the basin (B). Patterns: red  =  St. Paul; black  =  St. George; Years: 2008 =  dots, 2009  =  crossed lines, 2010  =  diagonal lines.

In all years combined, kittiwakes used shelf habitats more often (64%, *n* = 157 individual trips) than basin habitats (36%). Males foraged more often on the shelf in 2008 (10/14 trips) and 2009 (15/17 trips), but not 2010 (20/30 trips), when their trips to the basin increased by 66%. In contrast, females used both habitats with ∼ 42% of trips to the basin in 2008 (*n* = 12) and 2009 (*n* = 15), decreasing to 33% in 2010 (*n* = 33). Between colonies, birds from St. George (*n* = 82 trips) traveled more frequently to the basin than those from St. Paul (*n* = 75 trips; 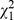 =  8.521, *P* = 0.003; 46%, *vs*. 24% respectively (Fig. 1A). Among years, birds from St. Paul foraged more often in the basin in 2010 than 2008 and 2009, whereas use of the basin was similar among years for birds from St. George (Fig. 1B). Nearshore (≤ 3 km from shore) foraging occurred mainly around St. Paul, and was greater in 2008 (76%, *n* = 29 trips) than 2009 (25%, *n* = 40 trips) or 2010 (45%, *n* = 89 trips, Fig. 1C). Core feeding areas, delineated by 50% contours, from trips to the shelf were near the islands, and in oceanic waters near or over the shelf break during trips to the basin in all years (Fig. 2). In oceanic waters, there were several core feeding areas, which showed some overlap (10–75%) among years at St. George, and also between colonies in 2009 and 2010 (5–10%, Fig. 2).

**Figure 3:**
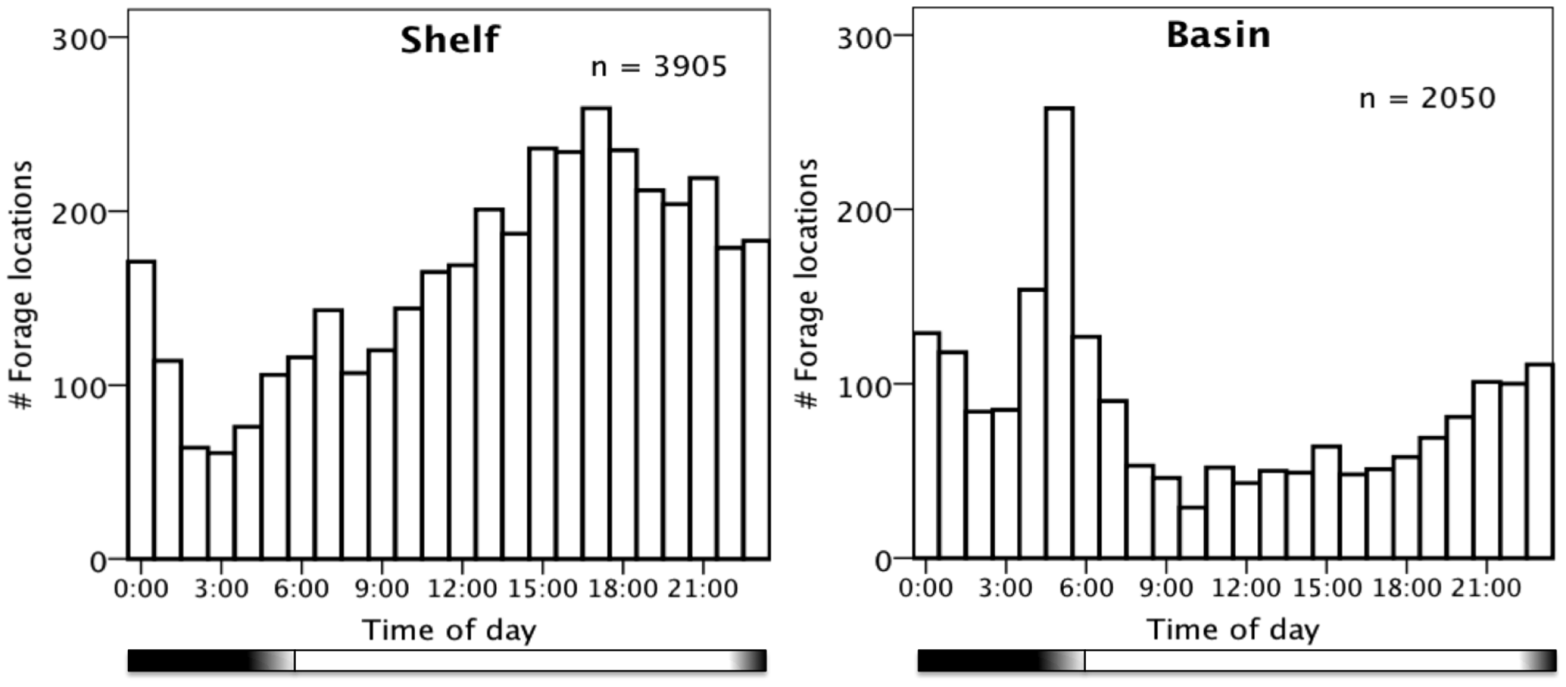
Frequency histograms of foraging locations of black-legged kittiwakes from St. Paul and St. George Islands according to time of day and marine habitat (shelf and basin) of the southeastern Bering Sea. The rectangle indicates the dark (black), twilight (grey), and daylight (white) periods.

Birds from both colonies showed similar distributions of foraging locations with regard to time of day. In the basin, birds foraged during all hours, but activity was greater (56%; *n* = 2050) during darkness and twilight (2300–0600h), with a peak during morning twilight (Fig. 3). In contrast, over the shelf, feeding activity peaked during 1400–1800h, and most foraging events (80%; *n* = 3905) occurred during daylight (0600–2200h).

On average, the maximum trip distances on the shelf for birds from St. Paul were 27.2**±**2.4 km (*n* = 57 trips), and for those from St. George were 30.2**±**2.9 km (*n* = 41 trips). Maximum distances for trips to the basin were much longer for both colonies (St. Paul 201.4**±**6.9 km, *n* = 18, St George 160.4**±**6.4, *n* = 37). In the full-factorial model, there was no significant interaction between sex and the other main factors (all tests *P*>0.05), but there was between colony and year (Table 3). Birds nesting at St. George traveled further distances on average than those at St. Paul in 2008 (*F*_1, 39_ = 13.415, *P* = 0.001), but not in 2009 (*F*_1, 77_ = 2.184, *P* = 0.144) and 2010 (*F*_1, 185_ = 0.176, *P* = 0.676). In 2010, trip distance was affected by both habitat (*F*_1, 183_ = 811.7, *P*<0.0001) and colony (*F*_1, 183_ = 7.317, *P* = 0.007). Birds from St. Paul (206.7±6.7 km) made significantly longer trips to the basin than those from St. George (169.3±10.3 km; *F*_1, 42_ = 7.148, *P* = 0.011) in 2010; however, they did not differ in trip distances on the shelf (St. Paul 25.1±2.5 km, St. George 31.6±3.7 km; *F*_1, 141_ = 2.464, *P* = 0.119; Table 4).

**Table 3:** Linear mixed model of trip distance of black-legged kittiwakes with colony (St. Paul  =  STP; St. George  =  STG), sex and year (2008, 2009, 2010) as fixed factors and individual as a random factor.

	Sum of Squares	*df*	*F*	*P*	Outcome
Colony	4.259	1	13.106	**<0.0001**	
Sex	0.222	1	0.683	0.409	
Year	1.003	2	1.543	0.215	
Colony*Sex	0.474	2	0.831	0.228	
Colony*Year	3.401	2	5.233	**0.006**	STG>STP (2008 only)
Sex*Year	1.662	2	2.557	0.079	
Colony*Sex*Year	1.319	2	2.030	0.133	
Error	95.873	295			

Statistically significant (*P* < 0.025 after Bonferroni correction) relationships are shown in bold (see text for post-hoc comparisons).

**Table 4:** Linear mixed model of trip distance of black-legged kittiwakes with colony (St. Paul  =  STP; St. George  =  STG) and habitat (Shelf and Basin) as fixed factors and individual as a random factor.

	Sum of Squares	*df*	*F*	*P*	Outcome
Colony	7565.1	1	7.317	**0.007**	Shelf: STP = STG
Habitat	839230.5	1	811.7	**<0.0001**	Basin: STP>STG
Colony*Habitat	16170.5	1	15.64	**<0.0001**	
Error	189203.5	183			

Statistically significant (*P* < 0.025 after Bonferroni correction) relationships are shown in bold (see text for post-hoc comparisons).

#### Association of foraging kittiwakes with basin eddies

**Figure 4:**
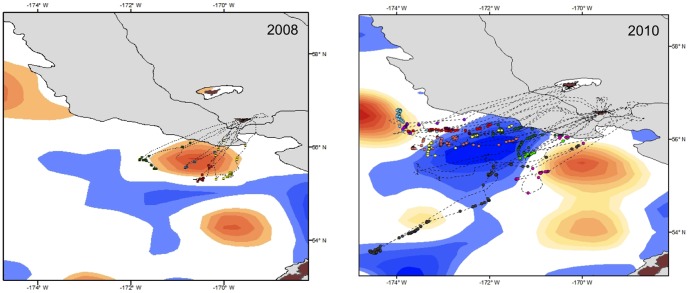
Examples of individual tracks and foraging locations (circles) overlaid on eddy fields (blue  =  cyclonic eddies, orange  =  anticyclonic; generated using AVISO Products) matched by date (July 23±7 days 2008; July 7±3 days 2010) of data collection.

**Figure 5:**
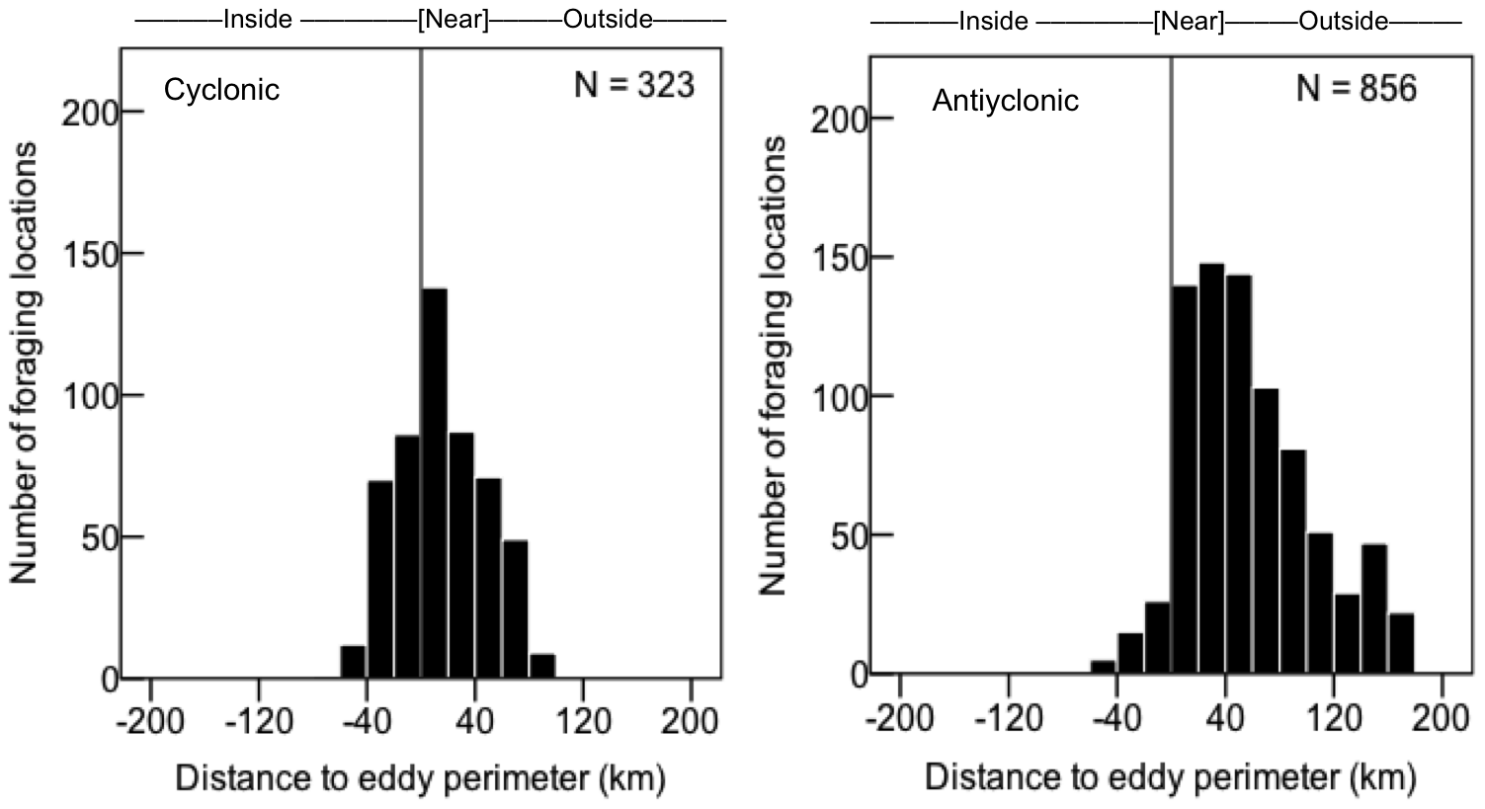
Frequencies of foraging and random locations according to distance to nearest eddy perimeter (vertical line); negative values indicate locations inside features.

Bird tracks and foraging locations overlapped with both anticyclonic and cyclonic eddies in the basin (Fig. 4). Frequencies of locations inside, near, and outside eddies differed between random and foraging locations, with foraging locations more associated with eddies than expected by chance (cyclonic: 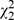 =  40.4; *P* < 0.001; anticyclonic: 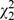 =  38.5; *P* < 0.001). Foraging locations were associated with both cyclonic and anticyclonic eddies; however, the association varied by colony and year, apparently due to the differing proximity and intensity of these features rather than a colony-specific preference. In 2008 and 2009, all foraging locations (*n* = 49) of kittiwakes from St. George were near or within anticyclonic eddies near the shelf break. Cyclonic meanders were farther from the shelf break in 2008 than 2010 (Fig 4). In these two years, the SSHA in anticyclonic eddies was lower than in 2010 (8–13 cm *vs.* 17–21 cm). In 2010, foraging locations of birds from St. Paul were more often near anticyclonic eddies (68%, *n* = 316 locations), whereas those of birds from St. George were more often near or inside cyclonic eddies (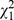 =  53.7; *P* < 0.0001; 83%, *n* = 139 locations). There were 3 eddies that persisted along the shelf break within the study area in 2010. From the eddy edges, the anticyclonic eddy with the highest SSHA (21 cm) was farther from both islands (211–243 km), but still 31 km nearer St. Paul than St. George. The second anticyclonic eddy (17 cm SSHA) was closer to both islands (123–145 km), and 21 km nearer to St. George than St. Paul. A cyclonic eddy (–9.7 cm SSHA) was the closest eddy to both islands (at 83–109 km), and was 26 km near St. George than St. Paul. There was a higher frequency of foraging locations inside and near (59%, *n* = 593), than outside the perimeters of cyclonic than anticyclonic eddies (21%, *n* = 856; 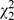 = 193.0; *P* < 0.0001). In contrast, half of the foraging locations adjacent to anticyclonic eddies were found within 60 km of the perimeter, and less than 10% were in the interior (Fig. 5).

### Diet and Habitat-prey Relationships

**Figure 6:**
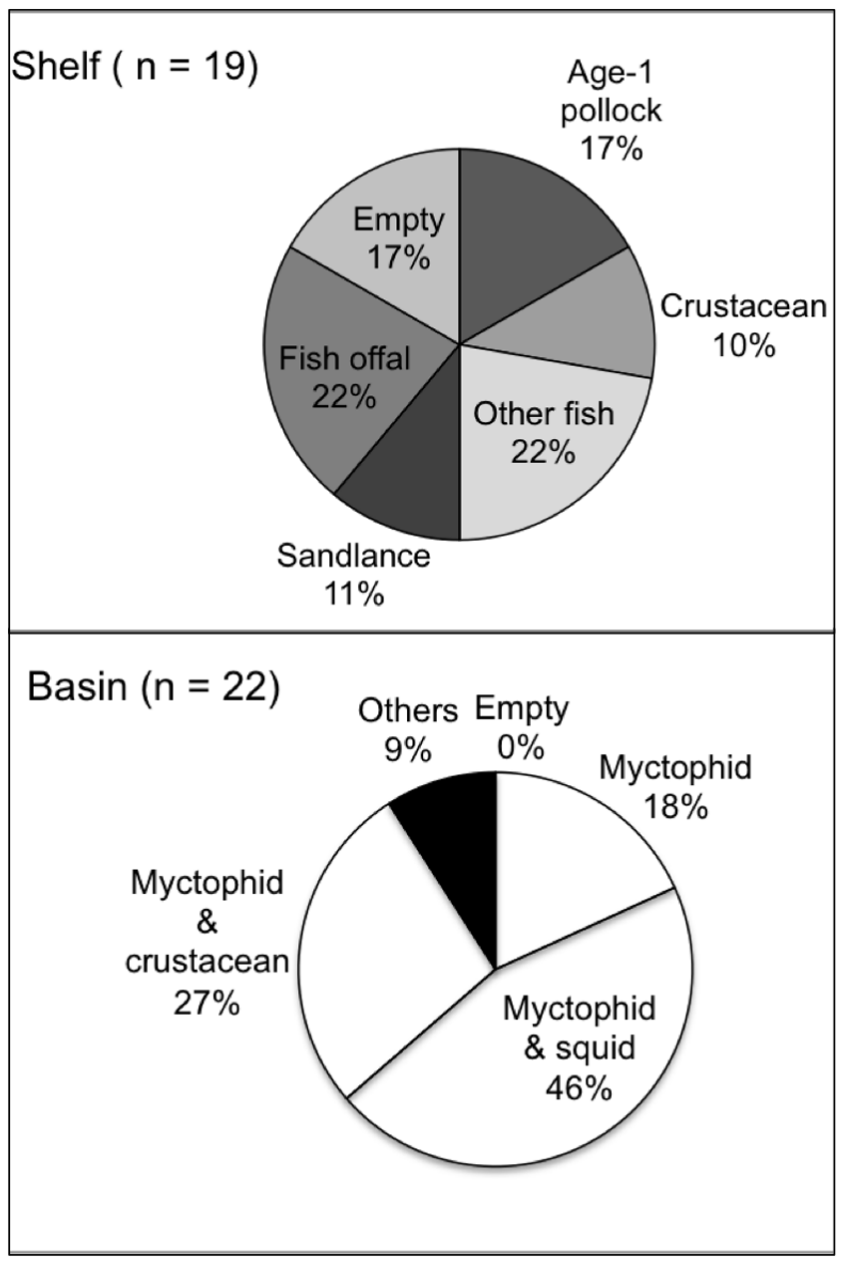
Prey species consumed by tracked kittiwakes during trips to shelf and basin habitats. Percentages are based on the total number of samples (*n*) of trips to each habitat. “Other fish”: eelpout, rockfish, and gadids; “Crustaceans”: amphipods and euphausiids; “Others”: amphipods and sea nettle. Empty samples from stomach lavages were only found from birds that traveled to the shelf in 2008.

Analysis of diet of tracked birds showed that age-1 pollock, sandlance, eelpouts (*Lycodes sp*.), rockfish (*Sebastes sp*.) and fish offal were obtained on the shelf (*n* = 19), whereas myctophids and squid (e.g. *Gonatopsis borealis*) were obtained primarily in oceanic habitat (*n* = 22; Fig. 6). *S. leucopsarus* (72–77%) was the most common myctophid species across years, and *S. nannochir* (16%) and *N. regale* (4%) were found mainly in 2010. Euphausiids (*Thysanoessa raschii*) and amphipods (*Themisto libellula*; *T. pacifica*) were obtained in combination with forage fish species either on the shelf or in the basin.

**Figure 7:**
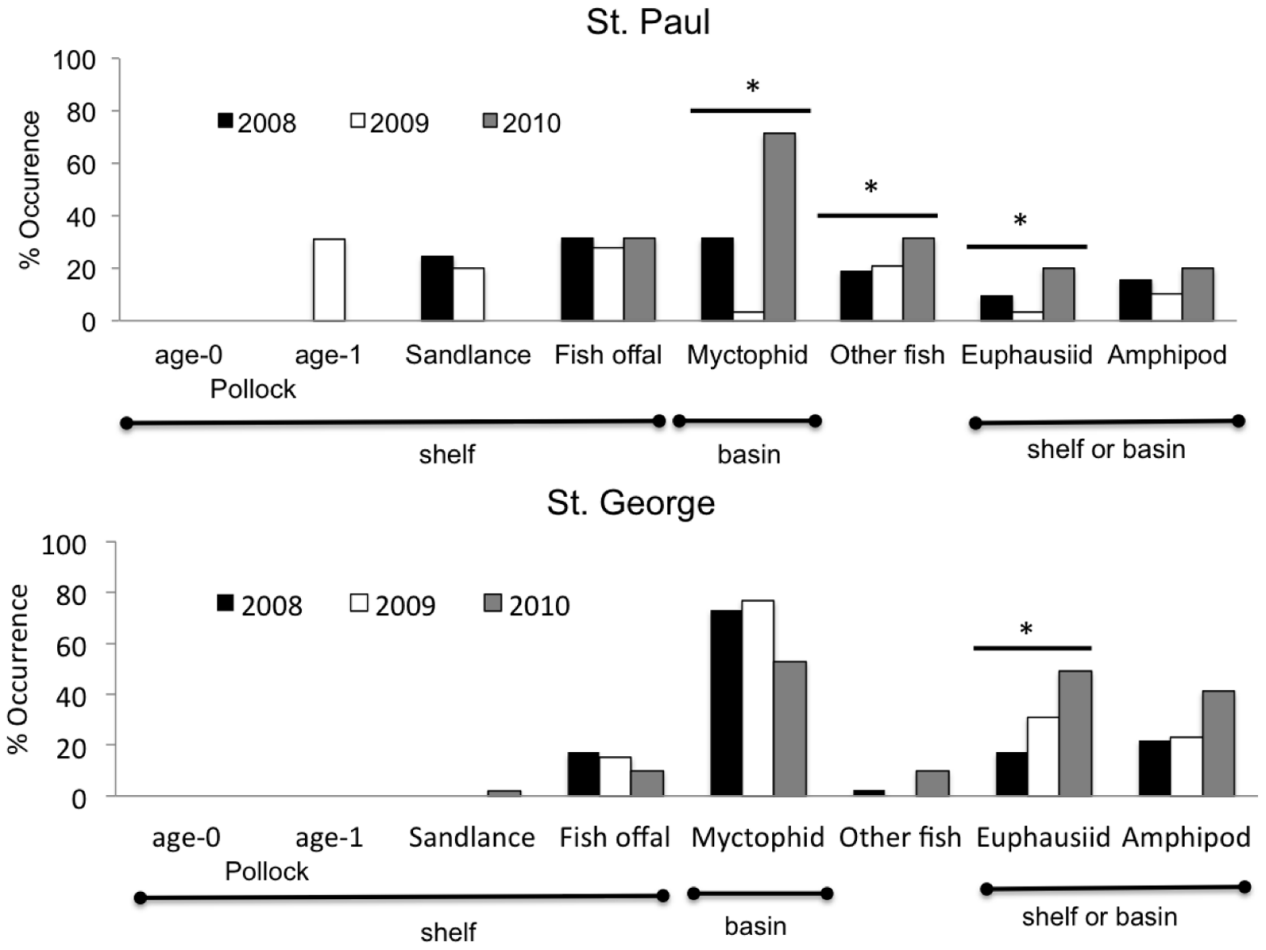
Diet composition of chick-rearing kittiwakes from St. George (*n* = 41 samples in 2008; *n* = 26 in 2009; *n* = 51 in 2010) and St. Paul Islands (*n* = 32 in 2008; *n* = 29 in 2009; *n* = 35 in 2010). Categorization of prey by domain location (shelf and basin) was based on diet of tracked birds (Fig. 6).

Diet analysis of all birds sampled showed that birds from St. George fed mainly on myctophids, and those from St. Paul on a greater variety of fish (Fig. 7). Myctophid and squid occurrences in kittiwake diets at St. George did not differ among years (all tests *P*>0.05, Fig. 7), whereas euphasiid (*H* = 11.980, *df* = 2, *P* = 0.003) and amphipod occurrences (*H* = 6.010, *df* = 2, *P* = 0.049) increased from 2008 to 2010. Myctophid (*H* = 28.89, *df* = 2, *P* < 0.0001), squid (*H* = 9.058, *df* = 2, *P* = 0.011) and euphasiid (*H* = 6.665, *df* = 2, *P* = 0.036), but not amphipod (*P* = 0.880) occurrences increased significantly in the diet at St. Paul over the three study years (Fig. 7). Shelf-based preys such as juvenile pollock were only present in the diet of kittiwakes in 2009 at St. Paul, when birds consumed age-1 pollock. Pacific sandlance was recorded at St. Paul in 2008 and 2009. Fish offal was common in diets at both colonies and its occurrence did not differ among years or colonies (all tests *P*>0.05; Fig. 7).

Analysis of longer-term data showed significant positive correlations between EKE and myctophid occurrence in the diet at St. Paul, and EKE and euphasiids at St. George (Table 5). Amphipod occurrence also increased with EKE when samples from both colonies were pooled; no relationships were found between EKE and squid occurrence (Table 5).

**Table 5:** Correlations between eddy kinetic energy (mean in July), prey occurrence in diets and productivity of black-legged kittiwakes nesting at the Pribilof Islands (6–10 year-data sets, see Appendix S1).

EKE vs.	St. Paul		St. George		Total	
	*R*	*P*-value	*R*	*P*-value	*R*	*P*-value
Prey						
Myctophids	+ 0.732	**0.031**	+0.151	0.349	+ 0.356	0.088
Euphasiids	+ 0.691	**0.043**	+ 0.770	**0.008**	+ 0.607	**0.006**
Amphipods	+ 0.563	0.094	+ 0.522	0.075	+ 0.491	**0.027**
Squid	– 0.452	0.154	– 0.073	0.426	– 0.123	0.325
Productivity						
Fledging success	+0.176	0.738	+0.216	0.578	+0.185	0.510

Significant relationships indicated in bold.

### Prey Availability

**Figure 8:**
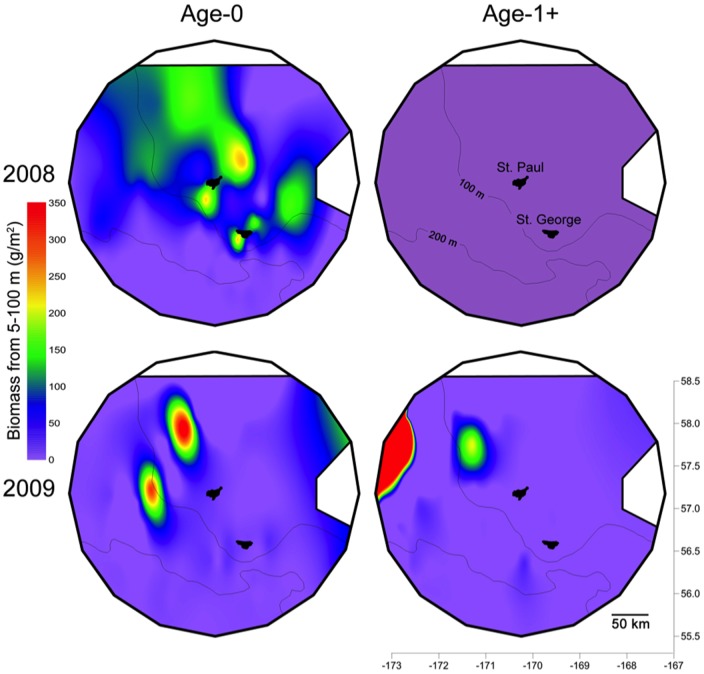
Acoustically measured biomass density (g/m^2^) of juvenile pollock integrated from 100 m to the surface. Data were collected from mid-July to mid-August in each year over 110 transects of 10 km in length that were randomly placed within 200 km of St. George Island. Map surfaces were generated using minimum curvature interpolation.

The average biomass density of juvenile pollock, including both young of year and age-1+, within 200 km of St. George Island was similar between the two years (2008: 43.4 g/m^2^, 95% CI 30.5–57.1; 2009: 41.7 g/m^2^, 95% CI 16.8–66.5). This biomass, however, showed contrasting distribution in space and across year classes. Age-1+ pollock were virtually absent in 2008, but occurred in two discrete patches northwest of St. Paul in 2009 (Fig. 8), the only year when kittiwakes consumed pollock. The distances from St. Paul Island to these patches were 174.8 –176.3 km (*n* = 2 transects) and 59.2 – 89.9 km (*n* = 3) for the large and small patches respectively (Fig. 8). Trips by kittiwakes from St. Paul to these patches were significantly longer than those to other directions (*F*_1, 35_ = 23.211, *P* < 0.001). Although age-0 pollock was present at sea in both years, kittiwakes did not feed on this prey (Fig. 7 & 8). The higher variance in pollock biomass density in 2009 than 2008 is indicative of the highly aggregated spatial distribution observed in juvenile pollock of both year classes within the study area (Fig. 8).

### Fitness Benefits

**Figure 9:**
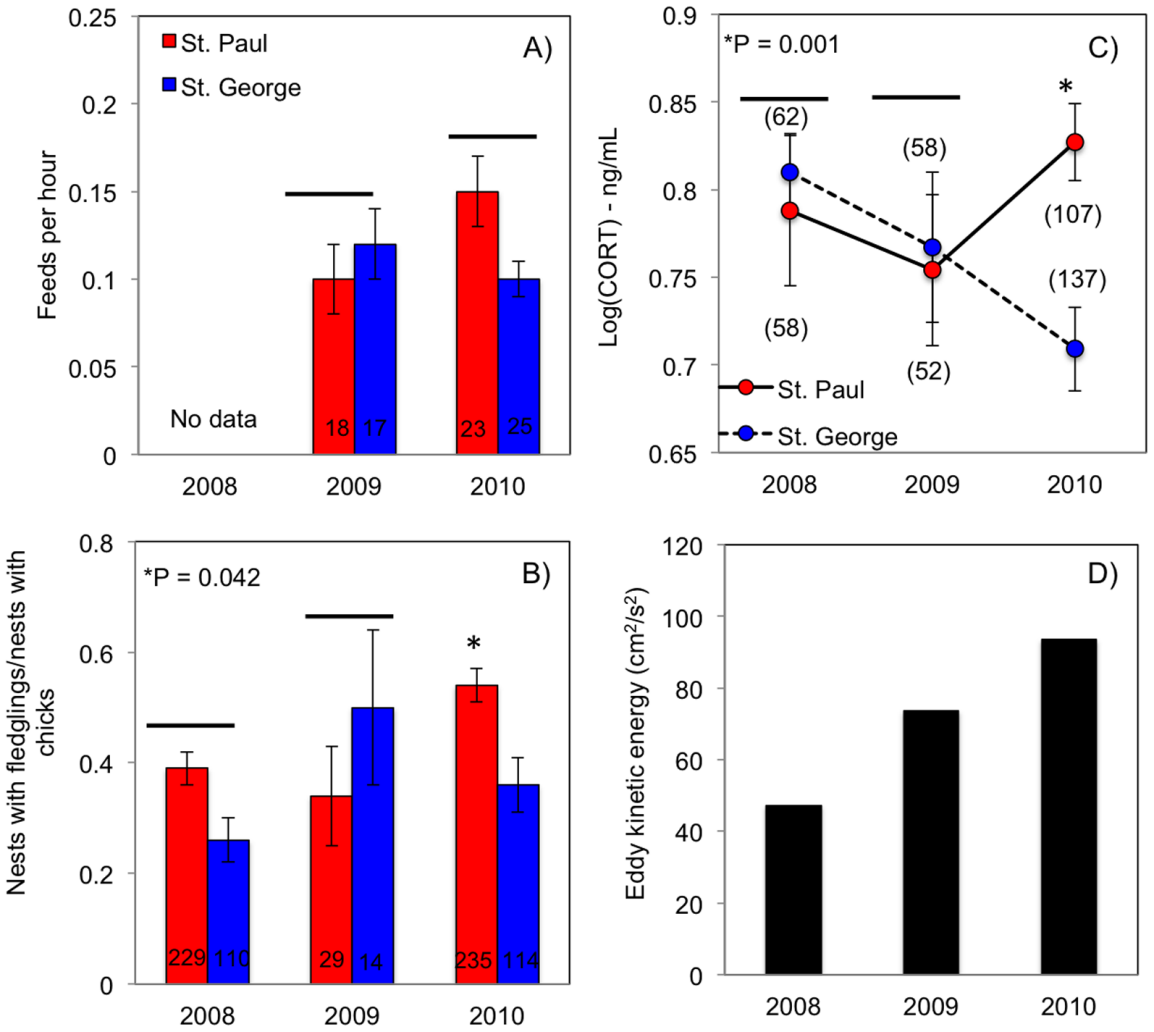
Breeding performance and physiological stress of black-legged kittiwakes at St. Paul and St. George islands, and eddy kinetic energy in the basin area during 2008-10. Chick-feeding frequency (A), fledging success (B), adult nutritional stress levels (C), and July-mean eddy kinetic energy (D). Decreasing stress levels of St. George birds coincide with the increase in EKE between 2008 and 2010.

Chick-feeding rates did not differ between colonies (*F*_1, 79_ = 0.458; *P* = 0.501) or years (*F*_1, 79_ = 0.877; *P* = 0.352; Fig. 9A), although the interaction approached statistical significance (*P* = 0.067). Fledging success showed a significant interaction between colony and year (*F*_2, 49_ = 3.394; *P* = 0.042); it did not differ between colonies in 2008 (*F*_1, 21_ =  1.411; *P* = 0.248) and 2009 (*F*_1, 11_ = 1.999; *P* = 0.185), but was higher at St. Paul than St. George in 2010 (*F*_1, 17_ = 4.827; *P* = 0.042; Fig. 9B). Fledging success varied among years (*F*_2, 52_ = 3.508; *P* = 0.037) and was higher in 2010 than 2009 (post-hoc test: *P* = 0.040) but did not differ significantly between 2008 and 2010, or 2008 and 2009 (all tests *P*>0.05). Analysis of historical data showed no correlations between EKE and fledging success (Table 5).

We found an interactive effect of colony and year on CORT levels (*F*_2, 367_ = 3.265; *P* = 0.039); thus, factors were analyzed independently. There were no differences between colonies in 2008 and 2009 (*P*>0.05), but birds at St. Paul had higher stress levels than those at St. George in 2010 (*F*_1, 190_ = 10.80; *P* = 0.001; Fig. 9C). Within colonies, there was no difference in the stress levels of birds from St. Paul among years (*F*_1, 156_ = 1.280; *P* = 0.280). CORT levels of birds from St. George decreased as EKE increased from 2008 to 2010 (Fig. 9D); however, differences among years were not significant (*F*_1, 211_ = 2.79; *P* = 0.068).

## Discussion

Based on historical diets, we hypothesized that changes in foraging conditions in the southeastern Bering Sea shelf for black-legged kittiwakes have caused shifts in habitat use that have directly affected population trends. To test this, over three years (2008–2010), we used a suite of data on the at-sea distribution, breeding performance and nutritional stress (a proxy of adult survival) of kittiwakes at the Pribilof Islands, where populations have declined (St. Paul) or remained stable (St. George) since the mid 1970s. As an index of foraging conditions in shelf and basin habitats, we compiled data on kittiwake diets, the biomass and distribution of juvenile pollock, and eddy kinetic energy. Kittiwake responses were analyzed according to two main factors (habitat and colony; Table 1), and used to propose possible mechanisms for the contrasting population trajectories.

### Bathymetric Habitats and Effects on Diet of Kittiwakes

The spatio-temporal distribution of kittiwakes reflected contrasting availability of forage fish between shelf and basin habitats. As expected from their location, kittiwakes at the Pribilofs, particularly at St. Paul Island, concentrated foraging on the shelf despite the relatively low biomass of juvenile pollock (Fig. 8). Only in 2009 did birds tracked from St. Paul make 30–50% longer trips to the northwest of the shelf to access patches of age-1 pollock, which are larger and of higher energy content than age-0 fish [93]. Although we cannot discount the possible differences in availability of local fishery discards, the high prevalence of fish offal (∼ 20% at both islands) in diets in all years - 30 times more frequent than that reported in other localities in Alaska [38]- suggests that these kittiwakes may have been forced to feed on alternative or less profitable prey. We also found support for different temporal availability of prey for shallow feeders between bathymetric habitats [20]; kittiwakes fed less often during darkness on the shelf than over the basin (Fig. 3). Increased nocturnal foraging activity of visual predators in deep basin habitats may be facilitated by the bioluminescent organs of some of their prey species, such as myctophids [88].

As expected, in years when the incidence of neritic forage fish in the diet was low (St. Paul) or nil (St. George), kittiwakes made extensive overnight trips to the basin. Here they fed on energy-dense prey (myctophids and euphasiids) [93] following predictions of central place and optimal foraging that individuals will only travel greater distances for high quality, predictable prey, or prey with lower handling costs [56], [64]. The direct flights to the basin where feeding was concentrated (Fig. 1 & 2) coupled with the high prevalence of myctophids in diets suggest oceanic prey were available for kittiwakes. This might have been especially true in 2010 when EKE was much higher and birds from St. Paul increased usage of the basin by 63%, compared to 2008. We found a higher frequency of kittiwake foraging locations near both cyclonic and anticyclonic mesoscale eddies than would have occurred by chance, which is a similar situation to that reported previously for northern fur seals around the Pribilofs [82], [63]. Although surface-feeding kittiwakes could have access to vertically migrating *S*. *leucopsaurus* and their prey (euphasiids) [4], they could not access non-migrant myctophid species (*S. nannochir* and *N. regale*) [29], [90]. Interestingly, these two non-migrant myctophids were found only in kittiwake diets in the year of high EKE (2010). Thus, availability of mesopelagic prey may be enhanced by eddies as they can have a major influence on the vertical structure of prey fields [31].

In 2010, when birds from both colonies foraged in the basin, there were differences between colonies in the usage of each eddy type and also dietary changes. Bird from St. Paul foraged more often near anticyclonic eddies (upwelling) and occurrence of myctophids increased, whereas those from St. George were more likely to visit cyclonic eddies (downwelling) and myctophid incidence slightly decreased. Instead, the incidence of euphasiids increased in St. George diets. There is some evidence from diets of tracked seabirds of the targeting of myctophids on the periphery of anticyclonic eddies [62], [55]. Acoustic measures of prey abundance found higher biomass of large zooplankton and fish along the edge of an anticyclonic eddy than both inside and outside [31]. In this study, we found a greater number of kittiwake foraging at locations near the perimeter of anticyclonic, and inside or near cyclonic eddies, which agree with the predicted high productivity of these features in the Bering Sea [57]. Differences between colonies in the usage of each eddy type were apparently driven by the differing proximity and intensity (SSHA) of eddies in each year. Our analysis of the long-term data showed a positive relationship between EKE and the prevalence of oceanic prey in kittiwake diets, although with some differences between colonies in prey type. The lack of correlation between fledging success and EKE, however, suggests that oceanic prey may only partially compensate kittiwakes for poor conditions in local, neritic waters.

### Colony and Density-dependent Effects on Kittiwake Foraging

Foraging can also be influenced by factors associated with colony location [66], and top-down density–dependent mechanisms [2], [54]. As anticipated from colony locations, trips to the basin were considerably longer for birds from St. Paul; and their effort allocation was not apparently compensated by high-quality prey as indicated by their greater nutritional stress levels. Interestingly, although depletion of resources should in theory start sooner around the larger St. George colony [2], [24], there were no differences between colonies in trip distance on the shelf ([66], this study).

The near complete segregation in foraging areas of birds from the two islands supports the theory that neighboring colonies partition resources, possibly as a result of different foraging strategies maintained over generations [36], or direct avoidance of in-situ competition [54], [89]. For instance, in 2009, birds from St. Paul made trips northwest of the island to feed on age-1 pollock, whereas birds from St. George traveled equivalent distances to the basin. In this context, trips to the basin were 3–5 times longer than expected by the proximity of St. George to the shelf break, and may have been influenced by inter-specific competition for myctophids. Red-legged kittiwakes (*Rissa brevirostris*) mainly feed on myctophids [80], and apparently forage in different areas from black-legged kittiwakes (Paredes unpublished data). The potential competitive effect of red- and black-legged kittiwakes is considerably higher at St. George than St. Paul given their relative population sizes [12]. Kittiwakes from St. Paul had a slightly higher incidence of myctophids in their diet and higher fledging success than those from St. George in 2010. This additional factor may in part explain why the long-term positive relationship between EKE and myctophids was significant only at St. Paul, and that for EKE and euphasiids at St. George. Together, these results suggest density-dependent mechanisms may play a secondary role in determining foraging behavior and prey acquisition patterns in chick-rearing kittiwakes from St. George.

### Costs and Benefits – Implications for Population Processes

In stochastic environments, long-lived seabird species may be willing to pay survival costs of reproduction when they are close to the threshold at which reproduction becomes profitable [25], [26]. Our results support these predictions in that adult kittiwakes at both colonies adjusted foraging effort to fledge chicks although with apparent longer-term fitness costs. Extensive trips to the basin (max. 465 km, mean 268 km), above those recorded for the species elsewhere [83], [52], allowed kittiwakes to access profitable and predictable prey. Nonetheless, feeding rates and fledging success were below those reported for other years [10], [51], [86]. In addition, adult nutritional stress at both colonies was apparently higher than that found in warm years [75]; analogous to trends found for thick-billed murres at the Pribilofs (*Uria lomvia*) [7]. Satterthwaite et al. (2012) predicted from relative stress levels that the survival of adult kittiwakes would be lower during cold years, including in our study period of 2008–10, compared to warm years (ca.83% vs. ca. 89%). Our CORT measurements in 2010 suggest that, at least in some cold years, kittiwakes breeding on St. Paul could be affected more strongly than those breeding on St. George (Fig. 9C), confirming that the colony location might exacerbate detrimental effects of food shortages [12], [66]. Re-sighting individually marked adult kittiwakes on the focal colonies between 2009 and 2013 [51], [86] provide further support for the differing consequences of food shortages; in 4 out of 5 years, including 2011, apparent survival was higher at St. George than St. Paul (Renner et al. unpublished data). Given that these results are preliminary and differences in re sighting effort need to be taken into account, we cannot yet conclude a direct differential effect of stress on adult mortality between colonies. Elevated CORT may also be functioning as an anti-stress mechanism for a long-lived seabird skipping reproduction or abandoning breeding when conditions are poor [95].

Our results provide support for our hypothesis that long-term reduction in abundance of juvenile pollock, and an increase in mesopelagic prey in kittiwake diets [68] indicates increased usage of distant basin habitats, negative effects on adult nutritional stress, and by inference reduced survival. In this context, geographic location rather than total breeding numbers might explain differences between colonies in foraging patterns and population trajectories. Although our study was limited to the chick-rearing period, similar effects of food supply and colony on foraging during other stages of reproduction are likely. Declines in kittiwake abundance near the Pribilof Islands since the 1980s [45] provide further evidence for a shift in foraging areas, and highlight long-term environmental changes in the southeastern Bering Sea shelf.

Predicted increases in global temperatures are expected to affect food webs in ice-associated ecosystems, and therefore likely to affect predator-prey dynamics on the eastern Bering Sea shelf [43], [17], [81]. Here, recent climate models have forecasted declines in juvenile pollock recruitment over the next 40 years [58]. Given that kittiwakes cannot totally compensate for prolonged food-shortages, we predict continued decline of populations at St. Paul and possibly also at St. George, at the point that density-dependent effects become increasingly important.

In conclusion, chick-rearing kittiwakes were able to increase foraging effort by traveling to distant basin habitats, and so had some capacity to buffer food-shortages in the vicinity of their colonies. Enhancement of oceanic prey availability by eddies seems to only partially compensate for increased foraging effort by the parents, which incur high nutritional stress and, by inference, have a lower survival probability. In the context of long-term dietary changes for kittiwakes at the Pribilofs, our results suggest increased use of oceanic habitats over the last 35 years. These shifts in foraging areas might have contributed to the population decline at St. Paul, possibly through reduced adult survival or breeding frequency, due to the greater distance from oceanic waters.

## Supporting Information

Appendix S1 **Data used for correlation analysis between eddy kinetic energy (EKE) of the basin-study area, percentage of occurrence (% Oc.) of oceanic prey species, and fledging success of black-legged kittiwakes breeding at St. Paul (STP) and St. George (STG) islands.**
(DOCX)
