# Supplementary material for: Foraging Responses of Black-Legged Kittiwakes to Prolonged Food-Shortages around Colonies on the Bering Sea Shelf
Source: PLoS One. 2014 Mar 26;9(3):e92520. doi: 10.1371/journal.pone.0092520 (PMC3966792; doi:10.1371/journal.pone.0092520)
Supplement: Appendix S1 — Data used for correlation analysis between eddy kinetic energy (EKE) of the basin-study area, percentage of occurrence (% Oc.) of oceanic prey species, and fledging success of black-legged kittiwakes breeding at St. Paul (STP) and St. George (STG) islands. (DOCX) [file pone.0092520.s001.docx]

Appendix S1. Data used for correlation analysis between eddy kinetic energy (EKE) of the basin-study area, percentage of occurrence (% Oc.) of oceanic prey species, and fledging success of black-legged kittiwakes breeding at St. Paul (STP) and St. George (STG) islands.

| Year | Island | EKE^a^ | # Diet^b^ samples | % Oc. Squid | % Oc. Amphipod | % Oc. Euphasiid | % Oc.^c^ Myctophid | # Nests^d^ | Fledging Success |
| --- | --- | --- | --- | --- | --- | --- | --- | --- | --- |
| 1993 | STG | 59.0 | 11 | 9.1 | 0.0 | 0.0 | 27.3 | 33 | 0.61 |
| 1994 | STG | 38.7 | 5 | 20.0 | 20.0 | 00.0 | 80.0 | 14 | 0.29 |
| 1995 | STG | 132.0 | 0 | NS | NS | NS | NS | 0 | 0.0 |
| 1996 | STG | 73.8 | 0 | NS | NS | NS | NS | 7 | 0.43 |
| 1997 | STG | 47.6 | 9 | 22.2 | 0.0 | 11.1 | 55.6 | 73 | 0.76 |
| 1998 | STG | 53.3 | 39 | 23.1 | 5.1 | 33.3 | 33.3 | 46 | 0.72 |
| 1999 | STG | 44.0 | 13 | 0.0 | 7.7 | 15.4 | 7.7 | 3 | 0.00 |
| 2000 | STG | 67.8 | 9 | 0.0 | 0.0 | 22.2 | 77.8 | 77 | 0.82 |
| 2001 | STG | 42.0 | 7 | 0.0 | 0.0 | 0.0 | 0.0 | 17 | 0.35 |
| 2002 | STG | 39.3 | 0 | ND | ND | ND | ND | 88 | 0.88 |
| 2003 | STG | 200.3 | 0 | ND | ND | ND | ND | 52 | 0.81 |
| 2004 | STG | 91.7 | 0 | ND | ND | ND | ND | 57 | 0.54 |
| 2005 | STG | 76.9 | 0 | ND | ND | ND | ND | 11 | 0.55 |
| 2006 | STG | 38.9 | 0 | ND | ND | ND | ND | 91 | 0.60 |
| 2007 | STG | 46.2 | 0 | ND | ND | ND | ND | 7 | 0.14 |
| 2008 | STG | 47.3 | 41 | 2.4 | 22 | 17.1 | 73.2 | 104 | 0.26 |
| 2009 | STG | 73.7 | 26 | 7.7 | 23.1 | 30.8 | 76.9 | 14 | 0.5 |
| 2010 | STG | 93.6 | 51 | 15.7 | 41.2 | 49.0 | 52.9 | 115 | 0.36 |
| 1993 | STP | 59.0 | 8 | 0.0 | 0.0 | 0.0 | 50.0 | ND | ND |
| 1994 | STP | 38.7 | 0 | ND | ND | ND | ND | ND | ND |
| 1995 | STP | 132.0 | 0 | ND | ND | ND | ND | ND | ND |
| 1996 | STP | 73.8 | 0 | NS | NS | NS | NS | 119 | 0.56 |
| 1997 | STP | 47.6 | 30 | 6.7 | 0.0 | 0.0 | 53.3 | 135 | 0.65 |
| 1998 | STP | 53.3 | 30 | 6.7 | 0.0 | 6.7 | 16.7 | 200 | 0.67 |
| 1999 | STP | 44.0 | 0 | ND | ND | ND | ND | 41 | 0.29 |
| 2000 | STP | 67.8 | 5 | 0.0 | 0.0 | 0.0 | 60 | 217 | 0.82 |
| 2001 | STP | 42.0 | 0 | NS | NS | NS | NS | 105 | 0.60 |
| 2002 | STP | 39.3 | 0 | NS | NS | NS | NS | 260 | 0.87 |
| 2003 | STP | 200.3 | 22 | 0.0 | 0.0 | 0.0 | 0.0 | 216 | 0.77 |
| 2004 | STP | 91.7 | 0.0 | NS | NS | NS | NS | 292 | 0.63 |
| 2005 | STP | 76.9 | 0.0 | NS | NS | NS | NS | 59 | 0.41 |
| 2006 | STP | 38.9 | 9 | 0 | 0 | 0 | 33.3 | 147 | 0.54 |
| 2007 | STP | 46.2 | 0 | NS | NS | NS | NS | 3 | 0.00 |
| 2008 | STP | 47.3 | 32 | 6.3 | 15.6 | 9.4 | 25 | 229 | 0.39 |
| 2009 | STP | 73.7 | 29 | 0 | 10.3 | 3.4 | 3.4 | 29 | 0.34 |
| 2010 | STP | 93.6 | 35 | 0 | 20 | 20 | 71.4 | 225 | 0.54 |

ND = no data suitable for analysis (e.g. diet samples collected during incubation)

NS= no samples were collected in that year.

^a^ EKE was calculated in July each year within a polygon constructed based on all tracking data collected between 2008 and 2010.

^a^ These samples were collected during the chick-rearing period.

^c^ Only years with samples containing >4 % occurrence of myctophids were used for relationships with EKE. We assumed diets without myctophids indicated no use of basin habitats given that this prey is only obtained in the basin (Paredes et al. 2012, Fig. 7).

^d^ Number of nests with chicks.
